# Targeting the c-Met/VEGFR Pathway to Boost *Nab*-Paclitaxel Efficacy in Gastric Cancer: Preclinical Insights

**DOI:** 10.3390/cells15030285

**Published:** 2026-02-03

**Authors:** Jennifer Huang, Quinn Kaurich, Md Sazzad Hassan, Urs von Holzen, Niranjan Awasthi

**Affiliations:** 1 Department of Surgery, Indiana University School of Medicine, South Bend, IN 46617, USA; jenhuang50@gmail.com (J.H.); qkaurich@iu.edu (Q.K.); hassansa@iu.edu (M.S.H.); uvonholz@iu.edu (U.v.H.); 2 Harper Cancer Research Institute, University of Notre Dame, South Bend, IN 46617, USA; 3 Goshen Center for Cancer Care, Goshen, IN 46526, USA; 4 School of Medicine, University of Basel, CH-4056 Basel, Switzerland

**Keywords:** gastric cancer, c-Met, VEGFR, merestinib, *nab*-paclitaxel

## Abstract

**Highlights:**

**What are the main findings?**

- Merestinib significantly enhances the antitumor activity of *nab*-paclitaxel in gastric adenocarcinoma (GAC), particularly in c-Met–high preclinical models.
- Combination therapy induces tumor regression, prolongs survival, and suppresses tumor cell proliferation, microvessel density, and oncogenic signaling.

**What is the implication of the main finding?**

- Dual targeting of the HGF/c-Met pathway and microtubule dynamics represents a promising therapeutic strategy for gastric adenocarcinoma.
- Given the frequent HGF/c-Met overexpression in GAC, this combination may improve treatment outcomes in selected patient populations.

**Abstract:**

Combination chemotherapy regimens are commonly employed to treat advanced gastric adenocarcinoma (GAC), yet median survival remains less than one year. *Nab*-paclitaxel has demonstrated significant antitumor activity in preclinical GAC models. Overexpression of growth factors and their receptors is prevalent in GAC and contributes to its pathophysiology, with aberrant activation of the HGF/c-Met pathway reported in up to 50% of patients. We hypothesized that merestinib, a small-molecule inhibitor of c-Met, Axl, and DDR1/2, would enhance the therapeutic response to *nab*-paclitaxel in GAC. In high c-Met–expressing MKN-45 peritoneal dissemination xenografts in female NOD/SCID mouse models, animal survival was 17 days in controls, 37 days with *nab*-paclitaxel (118% increase), 24 days with merestinib (41% increase), and 43 days with the combination (153% increase), demonstrating significantly enhanced survival compared with either monotherapy. In MKN-45 subcutaneous xenografts, tumor volumes in the control, *nab*-paclitaxel, merestinib, and combination groups were 503 mm^3^, 115 mm^3^, 91 mm^3^, and −9.7 mm^3^ (indicating tumor regression), respectively. In low c-Met-expressing SNU-1 xenografts, tumor volumes were 219 mm^3^, 105 mm^3^, 131 mm^3^, and 57 mm^3^, respectively. IHC analysis of tumor cell proliferation and microvessel density in MKN-45 tumors supported these findings. In vitro, *nab*-paclitaxel and merestinib each reduced cell proliferation in GAC-associated cells, with enhanced inhibitory effects when used in combination. In MKN-45 cells, merestinib increased the expression of pro-apoptotic proteins and decreased phosphorylation of c-Met, EGFR, IGF-1R, ERK, and AKT. These results indicate that combining merestinib with *nab*-paclitaxel may represent a promising therapeutic strategy to improve outcomes for patients with GAC.

## 1. Introduction

Gastric adenocarcinoma (GAC) is the fifth most common malignancy and the fourth leading cause of cancer-related deaths worldwide [1]. Although the global incidence and mortality of gastric cancer have declined, it continues to pose a significant health burden, particularly in Latin America, Asia, Central Europe, and Eastern Europe [2]. Despite advances in therapeutic strategies, the prognosis for GAC remains poor, primarily due to late-stage diagnosis, early local invasion, metastasis, and limited effective treatment options. In Western countries, up to two-thirds of GAC patients present with advanced disease, and even in Japan, where extensive screening programs are implemented, approximately 50% of patients are diagnosed at an advanced stage [3].

Noncurative chemotherapy is commonly used in advanced GAC, but median overall survival (OS) remains approximately 9–10 months [4]. Even among patients with localized GAC who are treated with preoperative chemotherapy followed by surgery, the 5-year OS rate is only 38% [5]. Historically, regimens such as ECF (epirubicin, cisplatin, and 5-fluorouracil) and its variants were widely used [6,7,8]. In 2016, the FLOT regimen (5-FU/leucovorin, oxaliplatin, and docetaxel) demonstrated superior OS (50 months) compared with ECF/ECX (35 months) in patients with locally advanced, resectable tumors, establishing FLOT as the standard perioperative treatment for resectable GAC [9]. This regimen is also widely applied in metastatic GAC. Meta-analyses indicate that second-line therapy modestly extends survival in patients who fail first-line chemotherapy, typically using taxanes and irinotecan, or targeted agents such as trastuzumab and ramucirumab [10,11,12]. However, median OS in this setting remains limited (3.6–10.9 months) [13,14,15]. Low response rates, chemoresistance, and treatment-related toxicity [16] underscore the urgent need for novel therapeutic approaches to improve GAC outcomes.

Several growth factor signaling pathways, including VEGF/VEGFR, HGF/c-Met, PDGF/PDGFR, and EGF/EGFR, are implicated in GAC tumorigenesis, progression, metastasis, and resistance [17]. Based on the oncogenic potential of the HER-2 and VEGFR pathways in GAC, trastuzumab and ramucirumab have been approved as targeted therapies [18,19]. Similarly, the HGF/c-Met signaling pathway contributes to cancer cell proliferation, survival, metastasis, and angiogenesis, with c-Met activation observed in approximately 10–50% of GAC patients [20,21,22]. *c-Met* amplification and protein overexpression are associated with aggressive disease, invasion, metastasis, and poor prognosis, highlighting c-Met as an attractive therapeutic target [23,24].

Despite the development of several c-Met inhibitors (foretinib, SAR125844, tivantinib, among others), clinical trials have yielded disappointing results due to limited efficacy and significant toxicity [25]. Other pathways, including Axl and discoidin domain receptor 1/2 (DDR1/2), also contribute to GAC progression with Axl associated with tumor aggressiveness and poor prognosis and DDR1/DDR2 promoting invasion and metastatic potential [26,27]. Thus, a therapeutic strategy simultaneously targeting c-Met, Axl, and DDR signaling is strongly justified.

Merestinib (LY2801653, Mer; Supplementary Figure S1) is a potent, orally bioavailable multi-kinase inhibitor that targets c-Met, Axl and DDR pathways, in addition to MST1R, ROS1, MKNK1/2, and FLT3. It has demonstrated robust antitumor and antiangiogenic activity in MET-amplified and MET-autocrine xenograft models. Its low IC_50_ values (2 nM for c-Met and Axl, 0.1 nM for DDR1, and 7 nM for DDR2) suggest high potency with potentially favorable tolerability [28,29,30].

Conventional chemotherapeutic agents are frequently limited by toxicity. Nanoparticle-based formulations, such as nanoparticle albumin-bound paclitaxel (*nab*-paclitaxel), offer improved permeability, retention, and safety. *Nab*-paclitaxel, a next-generation taxane, has demonstrated superior antitumor efficacy and an improved safety profile compared with solvent-based taxanes in both preclinical and clinical studies [31,32,33,34]. It is FDA-approved for breast cancer, non-small cell lung cancer (NSCLC), and pancreatic cancer [35], and has demonstrated promising antitumor activity in gastric cancer models [36,37]. Furthermore, *nab*-paclitaxel has demonstrated synergistic activity when combined with targeted therapies and immunotherapies [37,38,39,40,41,42].

Given the poor survival rates associated with current treatments for GAC, the complexity of signaling networks in its progression, and the role of c-Met/Axl/DDR pathways in GAC metastasis, this study aimed to evaluate the antitumor efficacy of merestinib alone and in combination with *nab*-paclitaxel in preclinical GAC models.

## 2. Materials and Methods

### 2.1. Reagents

*Nab*-paclitaxel was obtained from the pharmacy at the Goshen Center for Cancer Care (Goshen, IN, USA), and merestinib was purchased from Adooq Biosciences (Irvine, CA, USA). The cell proliferation reagent WST-1 was purchased from Roche Diagnostics (Indianapolis, IN, USA), and RPMI 1640 growth medium was obtained from Sigma Chemical Corporation (St. Louis, MO, USA).

### 2.2. Cell Culture

Human GAC cell lines KATO-III and SNU-1 were purchased from the American Type Culture Collection (ATCC, Manassas, MD, USA), while MKN-45 was obtained from Creative Bioarray (Shirley, NY, USA). Human gastric fibroblasts were purchased from ScienCell Research Laboratories (Carlsbad, CA, USA). All cell lines were authenticated by the respective suppliers and routinely tested for mycoplasma contamination using MycoAlert (InvivoGen, San Diego, CA, USA). Detailed cell line characteristics are provided in Supplementary Table S1. Cells were cultured in RPMI-1640 supplemented with 10% or 20% fetal bovine serum (FBS) at 37 °C in a humidified atmosphere containing 5% CO_2_. Human gastric fibroblasts were cultured in a specialized fibroblast medium.

### 2.3. Cell Viability Assay

Cell viability was assessed using the WST-1 assay. Cells (4000–5000 per well) were seeded in 96-well plates and allowed to attach overnight. Cells were then treated with *nab*-paclitaxel (0.001, 0.01, 0.1, and 1 μM), merestinib (0.01, 0.1, 1, and 10 μM) or their combination and incubated for 72 h. Subsequently, 10 μL of WST-1 reagent was added to each well, followed by a 2-h incubation. Absorbance at 450 nm was measured using a microplate reader to determine cell viability.

### 2.4. Western Blot Analysis 

Subconfluent monolayers of cells were treated with *nab*-paclitaxel and/or merestinib (10 μM each) for 16 h. Whole-cell lysates were prepared, separated by SDS-PAGE, and transferred to PVDF membranes (Bio-Rad, Hercules, CA, USA). Membranes were incubated overnight at 4 °C with primary antibodies against phospho-c-Met, c-Met, phospho-Axl, Axl, phospho-EGFR, EGFR, phospho-IGF-1R, IGF-1R, phospho-ERK, ERK, phospho-AKT, AKT, cleaved caspase-3, Bcl-2 and GAPDH (Cell Signaling Technology, Danvers, MA, USA). After washing, membranes were incubated with HRP-conjugated secondary antibodies and developed using enhanced chemiluminescence (ECL, Cell Signaling). Protein bands were quantified using ImageJ software (NIH version 1.53K).

Notably, the maximum concentration of *nab*-paclitaxel used in the 72-h proliferation assays was 1 μM, whereas higher concentrations (10 μM) were used only for short-term (16-h) treatments in Western blot analyses to assess acute signaling and apoptotic responses, under conditions that preserved cell morphology and yielded high-quality protein lysates.

### 2.5. Tumor Implant and In Vivo Studies

All animal experiments were conducted in accordance with Institutional Animal Care and Use Committee (IACUC) guidelines at Indiana University School of Medicine (South Bend, IN, USA). Female NOD/SCID mice (4–6 weeks of age) were obtained from Charles River Laboratories (Wilmington, MA, USA). Mice were allowed to acclimatize for at least one week prior to the initiation of experimental procedures. Mice were housed under standard pathogen-free conditions with controlled temperature, with ad libitum access to food and water.

For the MKN-45 xenograft model, 7.5 × 10^6^ cells were injected subcutaneously into the right flank. Ten days post-implantation, when tumors became measurable, mice were randomized (*n* = 5 per group) to receive PBS (control), *nab*-paclitaxel (10 mg/kg, twice weekly), merestinib (10 mg/kg, 5 days/week), or the combination via intraperitoneal injection for two weeks. Doses and treatment schedules were selected based on prior published studies and preliminary experiments. Tumor volume (V) was calculated twice weekly as V = ½ (Length × Width^2^). Mice were monitored regularly for general signs of toxicity, including changes in body weight and behavior, throughout the treatment period. At study termination, mice were euthanized, and tumors were collected for histological, immunohistochemical, and Western blot analyses. A similar protocol was applied for SNU-1 xenografts (10 × 10^6^ cells). Blinding was not performed during treatment administration or tumor measurement; however, survival analysis, tumor growth data analysis, and immunohistochemical quantification were conducted in a blinded manner without knowledge of group allocation.

### 2.6. Animal Survival Analysis

For survival studies, MKN-45 cells (10 × 10^6^) were injected intraperitoneally into female NOD/SCID mice to establish a peritoneal dissemination model. Ten days later, mice were randomized (*n* = 5 per group) to receive PBS (control), *nab*-paclitaxel, merestinib, or the combination for two weeks, as described for the subcutaneous model. Mice were monitored daily for toxicity and euthanized when moribund based on predefined criteria (e.g., >15% weight change, tumor burden, lethargy, inability to ambulate). Survival was recorded from treatment initiation until death.

### 2.7. Immunohistochemical (IHC) Analysis

Subcutaneous tumors were fixed in 4% paraformaldehyde, embedded in paraffin, and sectioned at 5 μm. After deparaffinization and antigen retrieval, sections were blocked and incubated overnight with primary antibodies against Ki67 (rabbit polyclonal, Abcam, Waltham, MA, USA) or endomucin (rat monoclonal, Millipore Sigma, Burlington, MA, USA). Sections were incubated with Cy3-conjugated secondary antibodies (Jackson ImmunoResearch Laboratories, West Grove, PA, USA), counterstained with DAPI (Invitrogen, Carlsbad, CA, USA), and visualized using an Olympus IX81 fluorescence microscope (Olympus Corporation, Tokyo, Japan) with a Hamamatsu Orca camera (Hamamatsu Photonics, Hamamatsu City, Japan) and cellSens Dimension software version 1.18 (Olympus). Ki67- or endomucin-positive cells were quantified in three to four different high-power fields (HPF) per sample, with normalization for exposure time.

### 2.8. Statistical Analysis

Statistical analyses were performed using GraphPad Prism 7.0 (San Diego, CA, USA). Group comparisons were made using two-tailed Student’s *t*-test or one-way ANOVA, as appropriate. Survival was assessed using nonparametric log-rank tests. Data are expressed as mean ± standard deviation (SD). Significance thresholds were defined as ns (*p* > 0.05), * *p* < 0.05, ** *p* < 0.01, *** *p* < 0.001, and **** *p* < 0.0001. Differences between monotherapy and combination groups were analyzed similarly. Power calculations were performed using GPower 3.1, confirming that *n* = 5 mice per group provided sufficient statistical power. Data distribution was assumed to be normal; no formal tests for normality were performed.

## 3. Results

### 3.1. Enhancement in Animal Survival

**Figure 1:**
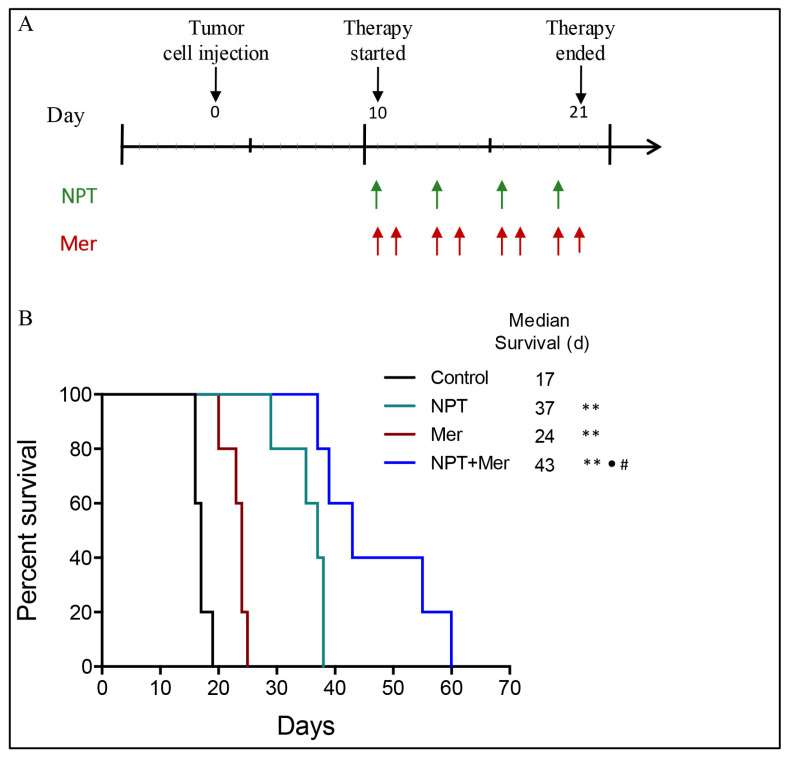
Survival analysis in MKN-45 cell-derived peritoneal dissemination xenografts established in female NOD/SCID mice. Ten days after tumor cell injection, mice were treated with *nab*-paclitaxel (10 mg/kg, twice weekly), merestinib (10 mg/kg, 5 days/week), or their combination for two weeks. (**A**) The upper panel illustrates the experimental design and the dosing schedule; arrows indicate days of drug administration. (**B**) The lower panel shows Kaplan–Meier survival curves from the start of treatment. Statistical differences in survival were calculated using the log-rank test. Significance levels are indicated as follows: control vs. treatment groups (** *p* < 0.01); *nab*-paclitaxel vs. combination therapy (^•^ *p* < 0.05), merestinib vs. combination therapy (^#^ *p* < 0.05).

The effect of merestinib and *nab*-paclitaxel on animal survival was evaluated using a peritoneal dissemination model of GAC established with MKN-45 cells (diffuse type, high c-Met expression) in female NOD/SCID mice. Given that peritoneal metastasis is common and associated with poor prognosis in GAC, this model has strong clinical relevance. Merestinib monotherapy significantly prolonged animal survival compared with control (24 days vs. 17 days; 41% increase, *p* = 0.002). *Nab*-paclitaxel monotherapy further improved survival (37 days; 118% increase, *p* = 0.002). Importantly, the combination of merestinib and *nab*-paclitaxel resulted in a significantly greater survival benefit, extending survival to 43 days (153% increase, *p* = 0.002 vs. control; *p* < 0.02 vs. either monotherapy) (Figure 1).

### 3.2. Reduction in Subcutaneous Tumor Growth

**Figure 2:**
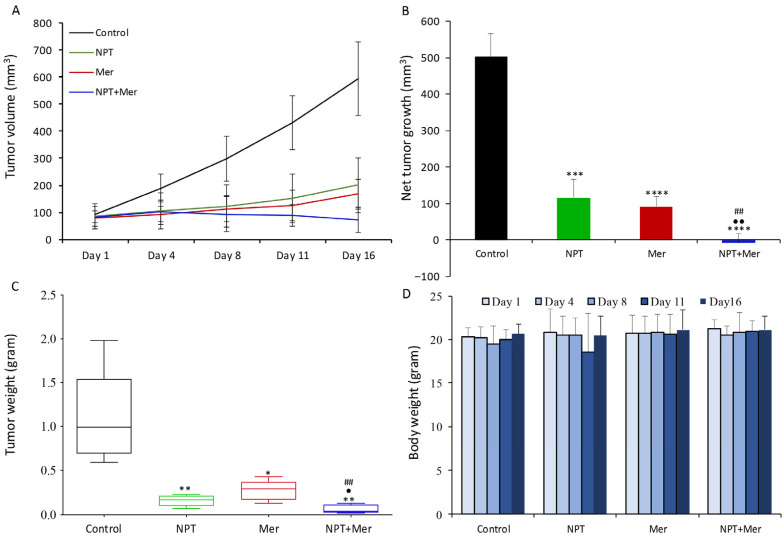
Tumor growth inhibition by merestinib and *nab*-paclitaxel in MKN-45 cell-derived subcutaneous xenografts established in female NOD/SCID mice: Ten days after tumor cell injection, mice were treated with *nab*-paclitaxel (10 mg/kg, twice weekly), merestinib (10 mg/kg, 5 days/week), or their combination for two weeks. (**A**) Tumor size was measured twice weekly using calipers and plotted over time. (**B**) Net tumor growth was calculated by subtracting tumor volume on the first treatment day from that on the final day. (**C**) At the end of the treatment, tumors were excised and weighed, and mean tumor weight is shown as a box plot. (**D**) Body weight was measured twice weekly during the treatment period and is presented as a bar graph. Statistical significance was determined using Student’s *t*-test: control vs. treatment groups (* *p* < 0.05; ** *p* < 0.01; *** *p* < 0.001; **** *p* < 0.0001); *nab*-paclitaxel vs. combination therapy (^•^ *p* < 0.05; ^••^ *p* < 0.01); and merestinib vs. combination therapy (^##^ *p* < 0.01). Data are presented as mean ± standard deviation from at least 5 mice per group.

The impact of merestinib and *nab*-paclitaxel on tumor growth was assessed in subcutaneous xenografts established in female NOD/SCID mice. In MKN-45 xenografts with high c-Met expression, control mice exhibited rapid tumor progression. Both *nab*-paclitaxel and merestinib monotherapies significantly reduced tumor growth (66% and 71% reduction, respectively). Combination therapy produced a significantly greater reduction in tumor size, achieving an 87.9% decrease (Figure 2A). Net tumor growth was 502.6 mm^3^ in controls, 115.3 mm^3^ with *nab*-paclitaxel, 90.6 mm^3^ with merestinib, and −9.7 mm^3^ with combination therapy, indicating tumor regression (Figure 2B). Mean tumor weights after 16 days of therapy were 1.001 g (control), 0.148 g (*nab*-paclitaxel), 0.254 g (merestinib), and 0.050 g (combination) (Figure 2C). These reductions paralleled tumor volume changes, supporting the antitumor efficacy of merestinib and its combination with *nab*-paclitaxel. Body weight remained consistent across treatment groups throughout the treatment period, with no significant weight loss observed, indicating an absence of overt treatment-related toxicity (Figure 2D).

**Figure 3:**
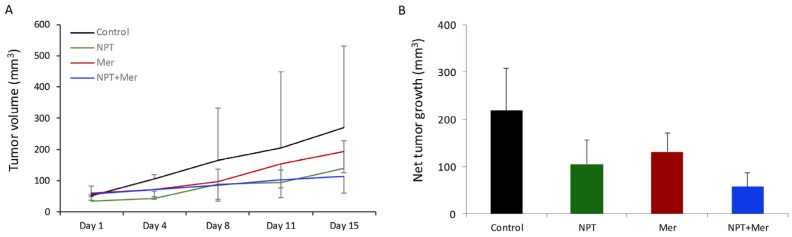
Tumor growth inhibition by merestinib and *nab*-paclitaxel in SNU-1 cell-derived subcutaneous xenografts established in female NOD/SCID mice: Ten days after tumor cell injection, mice were treated with *nab*-paclitaxel (10 mg/kg, twice weekly), merestinib (10 mg/kg, 5 days/week), or their combination for two weeks. (**A**) Tumor size was measured twice weekly using calipers and plotted. (**B**) Net tumor growth was calculated by subtracting tumor volume on the first treatment day from that on the final day. Data are presented as mean ± standard deviation.

In SNU-1 xenografts with low c-Met expression, monotherapy groups showed a trend toward reduced tumor volume and weight, while combination therapy showed a trend toward greater tumor reduction (Figure 3A,B). However, differences did not reach statistical significance, likely due to the limited number of animals per treatment arm. At the end of the therapy period, net tumor growth was 219.1 mm^3^ (control), 105.0 mm^3^ (*nab*-paclitaxel; 48% reduction), 131.3 mm^3^ (merestinib; 29% reduction), and 57.2 mm^3^ (combination; 58% reduction) (Figure 3B).

### 3.3. Decrease in Tumor Cell Proliferation and Microvessel Density

**Figure 4:**
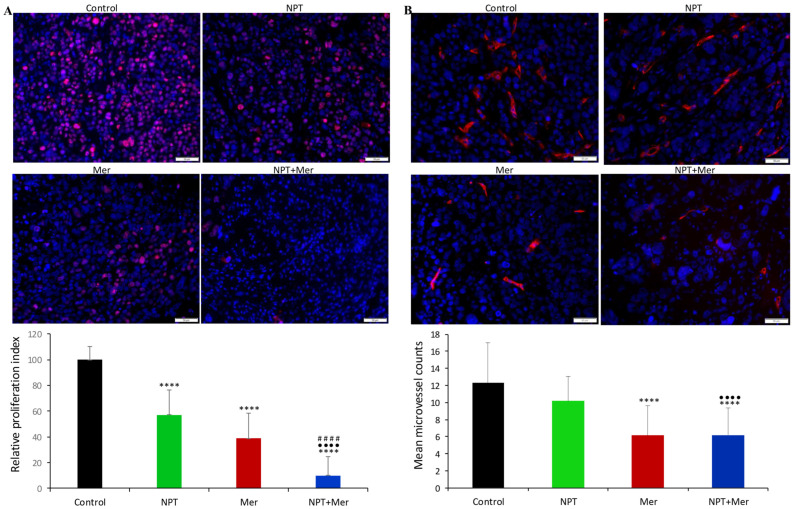
Effect of merestinib and *nab*-paclitaxel on tumor cell proliferation and microvessel density. Tumor sections from MKN-45 subcutaneous xenografts treated for two weeks with *nab*-paclitaxel (10 mg/kg, twice weekly), merestinib (10 mg/kg, 5 days/week), or their combination were analyzed by IHC. (**A**) Sections were stained for Ki67 (proliferation marker). (**B**) Sections were stained for endomucin (microvessel marker). Fluorescence microscope was used for visualization. Ki67- or endomucin-positive cells were quantified in three to four different high-power fields. Upper panels show merged images of cell nuclei (DAPI, blue) with Ki67 or endomucin staining (red) at 20× magnification. Statistical significance was assessed using Student’s *t*-test: control vs. treatment groups (**** *p* < 0.0001); *nab*-paclitaxel vs. combination therapy (^••••^ *p* < 0.0001); and merestinib vs. combination therapy (^####^ *p* < 0.0001). Data are presented as mean ± standard deviation (SD).

The biological effects of treatment were further examined in MKN-45 xenograft tissues. Tumor cell proliferation was evaluated by Ki67 staining. Both merestinib and *nab*-paclitaxel monotherapies reduced proliferation, while combination therapy achieved a markedly greater reduction. Relative to control (set at 100%), the intratumoral proliferative index (Ki67-positive cells/total cells per high-power field) was reduced to 57.0% with *nab*-paclitaxel (*p* < 0.0001 vs. control), 38.7% with merestinib (*p* < 0.0001 vs. control), and 10.0% with combination therapy (*p* < 0.0001 vs. control and vs. either monotherapy) (Figure 4A).

Considering the importance of angiogenesis in GAC progression and metastasis, we examined microvessel density in MKN-45 xenografts. Endomucin staining revealed that merestinib significantly decreased microvessel density, while *nab*-paclitaxel had no significant effect. Mean microvessel counts were 12.3 for control, 10.2 for *nab*-paclitaxel, 6.1 for merestinib, and 6.2 for combination therapy. Notably, combination therapy did not significantly differ from merestinib alone in reducing microvessel density (Figure 4B).

### 3.4. In Vitro Cell Proliferation Inhibition

In vitro cell proliferation assays were performed with multiple human GAC epithelial cell lines with different oncogenic mutations, as well as human gastric fibroblasts. Cell viability of human GAC cells, as well as human gastric fibroblasts, typically decreased with increasing concentrations of *nab*-paclitaxel and merestinib compared with controls.

**Figure 5:**
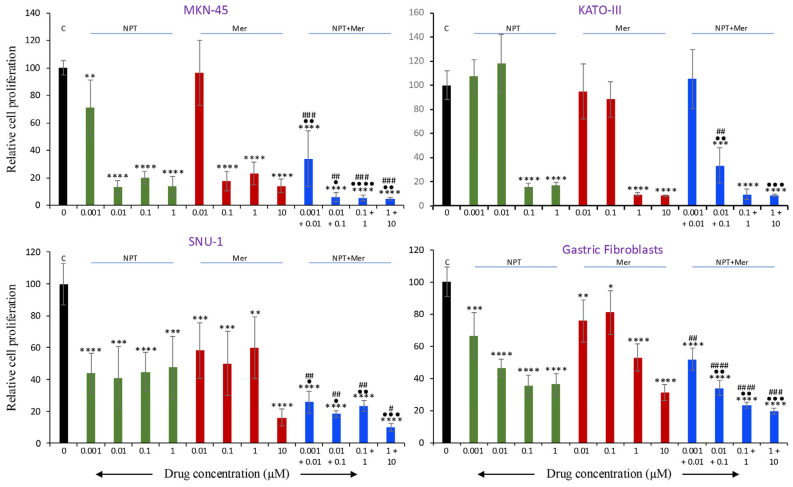
In vitro inhibition of cell proliferation by merestinib and *nab*-paclitaxel. GAC cell lines (MKN-45, SNU-1, KATO-III) and human gastric fibroblasts were seeded in 96-well plates and treated with *nab*-paclitaxel (0.001, 0.01, 0.1, and 1 μM), merestinib (0.01, 0.1, 1, and 10 μM), or their combination for 72 h. WST-1 reagent (10 μL) was then added to each well, followed by a 2-h incubation. Absorbance at 450 nm was measured to assess cell viability. Arrows indicate drug concentration on the x-axis; C denotes control. Green bars represent *nab*-paclitaxel (NPT), red bars represent merestinib (Mer), and blue bars represent combination treatment (NPT+Mer). Statistical significance was determined using Student’s *t*-test: control vs. treatment groups (* *p* < 0.05; ** *p* < 0.01; *** *p* < 0.001; **** *p* < 0.0001); *nab*-paclitaxel vs. combination therapy (^•^ *p* < 0.05; ^••^ *p* < 0.01; ^•••^ *p* < 0.001; ^••••^ *p* < 0.0001); merestinib vs. combination therapy (^#^ *p* < 0.05; ^##^ *p* < 0.01; ^###^ *p* < 0.001; ^####^ *p* < 0.0001). Data are presented as mean ± SD of quadruplicate determinations.

Merestinib decreased proliferation at 10 nM, 100 nM, 1 μM, and 10 μM by 4%, 82%, 77%, and 86% (MKN-45); 36%, 50%, 33%, and 84% (SNU-1); 5%, 12%, 91%, and 92% (KATO-III); and 24%, 19%, 47%, and 69% (gastric fibroblasts) (Figure 5). *Nab*-paclitaxel also showed dose-dependent inhibition at 1 nM, 10 nM, 100 nM, and 1 μM by 29%, 87%, 80%, and 86% (MKN-45); 52%, 59%, 52%, and 52% (SNU-1); −7%, −17%, 84%, and 83% (KATO-III); and 34%, 53%, 65%, and 64% (gastric fibroblasts) (Figure 5). Combination treatment resulted in greater inhibition of cell proliferation than either agent alone, reducing proliferation at the same doses by 66%, 94%, 95%, and 95% (MKN-45); 74%, 82%, 77%, and 90% (SNU-1); −5%, 66%, 91%, and 92% (KATO-III), and 48%, 66%, 77%, and 80% (gastric fibroblasts) (Figure 5).

To quantitatively assess drug–drug interactions, combination effects were analyzed using the Bliss independence model. In MKN-45 and KATO-III cells, the combination of NPT and Mer exhibited significant positive Bliss deviation at low concentrations, indicating synergistic inhibition of cell proliferation when single-agent activity was minimal. In contrast, SNU-1 cells and gastric fibroblasts displayed predominantly additive effects, with no consistent positive Bliss deviation across tested dose combinations. At higher concentrations in all cell types, where single-agent effects approached saturation, combination responses were additive or mildly antagonistic by Bliss criteria.

### 3.5. Effect on the Expression of Predictive Marker Proteins

**Figure 6:**
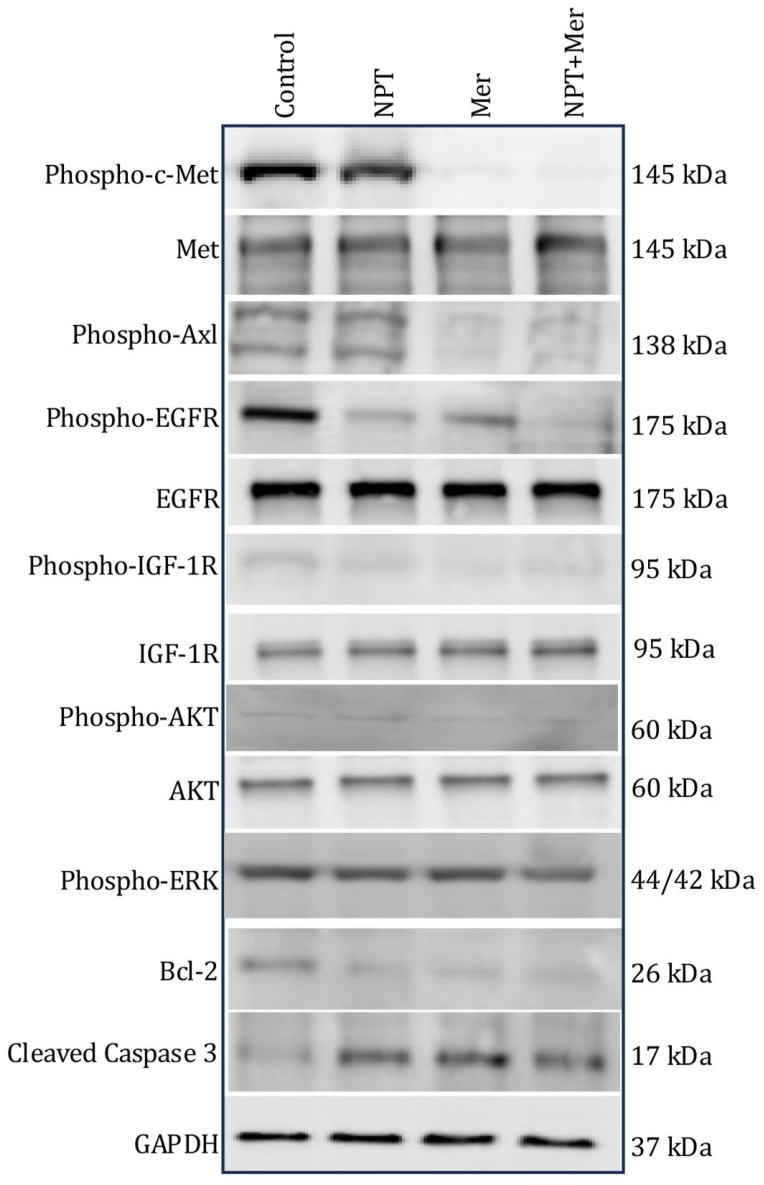
Expression of key oncogenic proteins in GAC cells following merestinib and *nab*-paclitaxel treatment. Subconfluent cultures of MKN-45 cells and human gastric fibroblasts were treated with merestinib (10 μM), *nab*-paclitaxel (10 μM), or their combination for 16 h. Whole cell lysates were prepared, and protein expression was analyzed by Western blot. Blots shown are representative of at least two independent experiments with consistent results.

To explore mechanisms underlying merestinib’s antitumor activity, we analyzed the expression of key oncogenic and apoptotic markers. In MKN-45 cells with high c-Met expression, merestinib, alone and in combination with *nab*-paclitaxel, increased the expression of apoptosis marker protein cleaved caspase-3. Merestinib decreased the expression of the anti-apoptotic protein Bcl-2 and reduced phosphorylation of c-Met, Axl, EGFR, IGF-1R, ERK and AKT (Figure 6).

## 4. Discussion

Although the incidence and mortality of GAC have declined in many developed countries, recent studies report an alarming rise among younger adults (<50 years) [1,43]. Systemic therapies for GAC, including chemotherapy, targeted therapy, and immunotherapy, have evolved significantly, with ongoing research exploring their potential benefits in the perioperative and adjuvant settings. For metastatic disease, progress has been made with biomarker-directed therapies, particularly those targeting PD-L1 and HER2 [44,45]. Nevertheless, intratumoral and intertumoral heterogeneity, late-stage presentation, and treatment resistance continue to drive poor outcomes.

Molecularly targeted approaches have demonstrated clinical benefit in GAC, exemplified by trastuzumab and ramucirumab, which validate HER2 and VEGFR2 as actionable drivers [18,19]. However, additional oncogenic pathways—including c-Met, Axl, and DDR—are implicated in tumor progression, angiogenesis, and metastasis. Importantly, prior clinical trials of selective c-Met inhibitors yielded disappointing results, likely due to heterogeneous c-Met expression, adaptive resistance, and narrow target specificity [25]. These limitations suggest that broader, multi-targeted strategies may be necessary to achieve durable efficacy.

Given its broad kinase inhibitory profile, merestinib’s antitumor activity cannot be ascribed exclusively to c-Met inhibition. While aberrant c-Met signaling is a well-established driver of GAC growth, invasion, and angiogenesis, additional targets of merestinib—particularly Axl and DDR1/2—also play critical roles in tumor progression and resistance. Axl signaling promotes survival, migration, epithelial-to-mesenchymal transition, and therapeutic resistance, while DDR1/2 activation through collagen–tumor interactions supports tumor cell adhesion, invasion, and pro-survival signaling within the tumor microenvironment [26,27]. Simultaneous inhibition of these pathways may limit compensatory signaling mechanisms that undermine selective c-Met blockade. Therefore, the enhanced efficacy observed with merestinib plus *nab*-paclitaxel likely reflects combined suppression of multiple oncogenic pathways, consistent with the heterogeneous and redundant signaling networks characteristic of GAC.

In addition to kinase-driven signaling pathways, accumulating evidence indicates that ion channels play critical roles in GAC cell proliferation, survival, apoptosis, angiogenesis, and therapeutic resistance. Dysregulated calcium (Ca^2+^), potassium (K^+^), and chloride (Cl^−^) channels contribute to GAC progression by modulating intracellular signaling, cell cycle regulation, and apoptotic thresholds [46,47]. Several oncogenic pathways targeted by merestinib—including c-Met, Axl, DDR, and downstream PI3K/AKT and MAPK signaling—are known to indirectly regulate ion channel expression and activity. For example, c-Met and Axl signaling can enhance calcium influx through store-operated calcium channels and transient receptor potential (TRP) channels, promoting tumor cell proliferation and migration [48,49], while PI3K/AKT-mediated modulation of potassium channels has been linked to apoptosis resistance and chemoresistance [50]. In parallel, microtubule-targeting agents such as *nab*-paclitaxel have been reported to disrupt intracellular calcium homeostasis and mechanosensitive ion channel function, contributing to mitotic arrest and cell death [51]. Thus, the enhanced antitumor efficacy observed with merestinib plus *nab*-paclitaxel may, at least in part, reflect indirect modulation of ion channel–dependent signaling networks downstream of kinase inhibition and cytoskeletal disruption. Although ion channel expression or activity was not directly assessed in this study, these pathways represent a plausible additional layer of therapeutic vulnerability in GAC that warrants focused investigation in future mechanistic and translational studies.

Merestinib is a potent inhibitor of c-Met, Axl, and DDR signaling, as well as other oncogenic kinases. Its broad target profile, combined with low nanomolar IC_50_ values, positions it as a promising therapeutic agent with potentially favorable tolerability. *Nab*-paclitaxel, meanwhile, improves chemotherapy delivery via enhanced permeability and retention effects and is known for anti-stromal and anti-mitotic activity [33,52]. Together, these mechanisms provide a strong biological rationale for combination therapy.

Our study demonstrates that merestinib and *nab*-paclitaxel each exert significant antitumor activity, and their combination produces greater antitumor effects than either agent alone. In MKN-45 xenografts with high c-Met expression, combination therapy not only inhibited tumor growth but induced regression, with parallel reductions in tumor weight. Even in SNU-1 xenografts with low c-Met expression, combination therapy resulted in numerically greater antitumor effects, suggesting that merestinib’s efficacy may extend beyond c-Met inhibition to include Axl, DDR1, MST1R, ROS1, MKNK1/2, and FLT3. Mechanistically, merestinib reduced tumor cell proliferation and angiogenesis, as evidenced by decreased Ki67 and endomucin staining in MKN-45 xenografts.

For in vitro cell proliferation assays of GAC cell lines, the quantitative Bliss independence analysis demonstrates that NPT–Mer synergy is dose-dependent and cell-line-specific, occurring primarily at low drug concentrations in MKN-45 and KATO-III cells. In contrast, SNU-1 cells and gastric fibroblasts showed predominantly additive responses, suggesting that synergistic interactions are not universal across gastric cancer models and are absent in non-malignant cells. At higher concentrations, combination effects were additive, consistent with saturation of single-agent activity. These findings support a selective synergistic interaction in responsive tumor cells, while preserving minimal effects in normal fibroblasts. Further, in vitro Immunoblot analysis demonstrated that merestinib modulated key oncogenic and apoptotic markers, decreasing phosphorylation of c-Met, Axl, EGFR, IGF-1R, and AKT, while increasing pro-apoptotic proteins.

We acknowledge that this study did not include genetic knockdown experiments to directly interrogate the contribution of individual merestinib targets. Given the broad kinase inhibitory profile of merestinib and the redundancy of oncogenic signaling networks in GAC, future studies incorporating targeted and combinatorial knockdown approaches will be important to further define mechanism and to support clinical translation of the merestinib–*nab*-paclitaxel combination.

Peritoneal dissemination is a particularly lethal manifestation of GAC [53]. In our peritoneal dissemination model using high phospho-c-Met–expressing MKN-45 cells, which closely mimics clinical disease progression, control mice showed extensive tumor spread to multiple organs [54]. Both *nab*-paclitaxel and merestinib monotherapies significantly improved survival, while combination therapy further enhanced survival, underscoring the therapeutic relevance of this approach for advanced disease. Although the combination regimen demonstrated robust antitumor efficacy and survival benefit, the present study did not include comprehensive systemic toxicity assessments such as hematologic or organ-specific analyses. Nevertheless, treatment was administered at doses below reported maximum tolerated doses for both *nab*-paclitaxel and merestinib, and no significant body weight loss or overt toxicity was observed during the treatment period. Future studies incorporating detailed toxicity profiling will be important to more fully define the therapeutic window and translational potential of this combination.

Taken together, our findings support merestinib as a multi-targeted therapy with activity against multiple oncogenic drivers of GAC. By simultaneously inhibiting tumor growth, proliferation, and angiogenesis and by enhancing chemotherapy efficacy, this strategy may overcome limitations of prior single-agent therapies. These preclinical results provide a rationale for clinical evaluation of merestinib plus *nab*-paclitaxel, ideally in biomarker-enriched patient populations (e.g., c-Met–overexpressing tumors). Future studies may also explore rational combinations with immunotherapy, given merestinib’s potential effects on the tumor microenvironment. From a translational perspective, our findings suggest that c-Met expression may represent a clinically relevant predictive biomarker for patient selection in merestinib-based combination therapy. The pronounced antitumor efficacy observed in c-Met–high MKN-45 models, compared with more modest effects in c-Met–low SNU-1 tumors, mirrors prior clinical experience indicating that unselected patient populations may dilute the benefit of c-Met–targeted therapies. These results support a biomarker-enriched strategy in which patients with c-Met overexpression or activation are prioritized for merestinib-based treatment. In the clinical setting, the merestinib plus *nab*-paclitaxel combination could be positioned within *nab*-paclitaxel–containing treatment strategies that have demonstrated clinical activity in advanced or metastatic GAC, particularly in the second-line or later-line setting, where *nab*-paclitaxel has been evaluated and used in clinical practice despite the absence of a formal regulatory approval for this indication. Systematic reviews and meta-analyses have reported meaningful antitumor activity and an acceptable safety profile for *nab*-paclitaxel monotherapy in previously treated GAC, supporting its consideration as a chemotherapy backbone in combination approaches [36]. Given the manageable toxicity observed in preclinical models and the established clinical tolerability of *nab*-paclitaxel, this combination may offer a rational approach to enhancing therapeutic efficacy without substantially increasing treatment-related burden. Prospective clinical evaluation incorporating biomarker-driven patient selection will be essential to define the optimal clinical context and therapeutic window for this strategy.

The selection of *nab*-paclitaxel as the chemotherapeutic backbone in this study was guided by translational relevance rather than an assumption of superior intrinsic cytotoxicity compared with solvent-based paclitaxel. Although formulation-specific advantages of *nab*-paclitaxel may be attenuated under standard serum-containing in vitro conditions, its use in cell-based assays ensured consistency with the in vivo treatment regimen and enabled mechanistic assessment of paclitaxel activity using its clinically relevant formulation. Clinically, in the phase III ABSOLUTE trial, weekly *nab*-paclitaxel was non-inferior to solvent-based paclitaxel in previously treated advanced gastric cancer, with numerically favorable progression-free survival and objective response rates but no overall survival advantage in the unselected population [15]. Importantly, post hoc analyses demonstrated improved survival with *nab*-paclitaxel in patients with peritoneal metastasis, a disease context directly modeled in our peritoneal dissemination studies. In addition to this potential disease-specific benefit, *nab*-paclitaxel offers practical clinical advantages, including avoidance of solvent-related hypersensitivity reactions, elimination of steroid premedication, shorter infusion times, and improved tumor penetration [34], supporting its evaluation in combination strategies for advanced GAC.

In parallel with targeted and cytotoxic therapies, there is growing interest in natural products and traditional medicine-derived compounds as complementary approaches in GAC. Several bioactive phytochemicals, including curcumin, resveratrol, berberine, and ginsenosides, have been reported to modulate growth factor–related signaling pathways relevant to GAC progression, including c-Met, EGFR, VEGFR, and downstream PI3K/AKT and MAPK cascades [55,56,57]. Preclinical studies further suggest that some of these agents may enhance sensitivity to chemotherapy and suppress angiogenesis and epithelial–mesenchymal transition. Although the clinical relevance of traditional medicine–based therapies remains to be fully established, these observations underscore the vulnerability of convergent oncogenic signaling networks in GAC and provide broader context for combination strategies targeting growth factor pathways.

Overall, our findings suggest that merestinib, particularly in combination with *nab*-paclitaxel, may improve the clinical management of GAC, especially in patients with c-Met overexpression. These results provide a strong preclinical rationale for further clinical evaluation of this therapeutic strategy.

## Data Availability

The data supporting the findings of this study are available within the article and its Supplementary Materials. Additional datasets are available from the corresponding author upon reasonable request.

## Supplementary Materials

The following supporting information can be downloaded at https://www.mdpi.com/article/10.3390/cells15030285/s1, Figure S1: Chemical structure of merestinib; Table S1: Characteristics of the human GAC cell lines used in the study.

## Abbreviations

The following abbreviations are used in this manuscript

GAC	gastric adenocarcinoma
NPT	*nab*-paclitaxel
Met	mesenchymal–epithelial transition factor
Mer	merestinib
VEGFR	vascular endothelial growth factor receptor
