# Supplementary material for: Targeting the c-Met/VEGFR Pathway to Boost Nab-Paclitaxel Efficacy in Gastric Cancer: Preclinical Insights
Source: Cells. 2026 Feb 3;15(3):285. doi: 10.3390/cells15030285 (PMC12897347; doi:10.3390/cells15030285)
Supplement: Supplementary file 1 [file cells-15-00285-s001.zip › Supplimentary Table 1.pdf]

Scheme 1. Characteristics of the human GAC cell lines used in the study.

GAC Cells	Origin	Differentiation	Histological type	Oncogene Expression
MKN-45	Liver metastasis	poorly	diffuse	c-met +; Ecad +
KATO-III	Pleural effusion	signet ring	diffuse	c-met +; FGFR2/K-sam+
SNU-1	Primary tumor	poorly	diffuse	myc +; erb B2 +
