# Supplementary figures and images for: Targeting the c-Met/VEGFR Pathway to Boost Nab-Paclitaxel Efficacy in Gastric Cancer: Preclinical Insights
Source: Cells. 2026 Feb 3;15(3):285. doi: 10.3390/cells15030285 (PMC12897347; doi:10.3390/cells15030285)

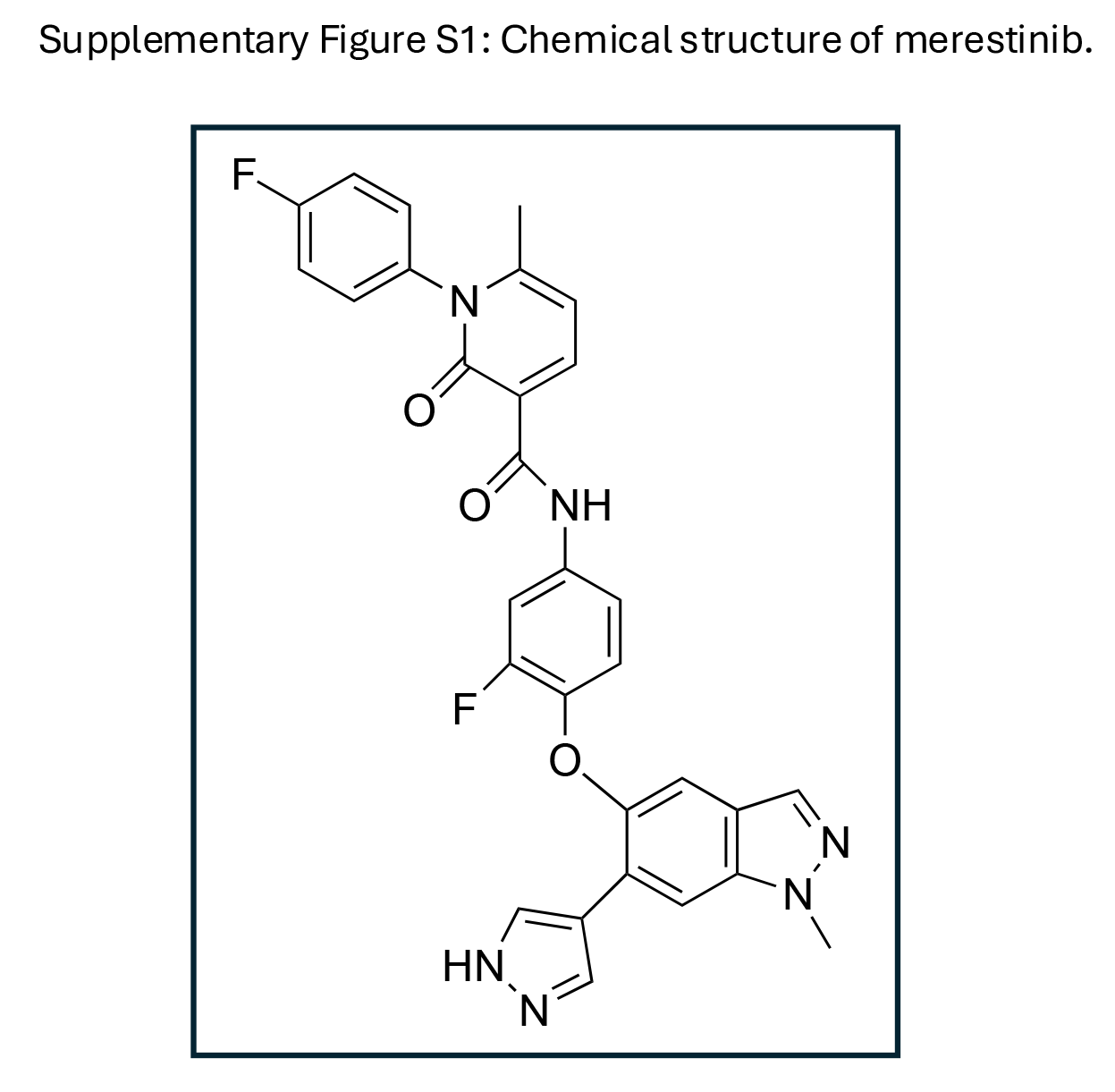

Supplement: Supplementary file 1 [file cells-15-00285-s001.zip › Supplementary Figure 1.tif]
